# Characterization of a Novel Metal-Dependent D-Psicose 3-Epimerase from *Clostridium scindens* 35704

**DOI:** 10.1371/journal.pone.0062987

**Published:** 2013-04-30

**Authors:** Wenli Zhang, Dan Fang, Qingchao Xing, Leon Zhou, Bo Jiang, Wanmeng Mu

**Affiliations:** 1 State Key Laboratory of Food Science and Technology, Jiangnan University, Wuxi, People’s Republic of China; 2 Roquette America, Keokuk, Iowa, United States of America; University of South Florida College of Medicine, United States of America

## Abstract

The noncharacterized protein CLOSCI_02528 from *Clostridium scindens* ATCC 35704 was characterized as D-psicose 3-epimerase. The enzyme showed maximum activity at pH 7.5 and 60°C. The half-life of the enzyme at 50°C was 108 min, suggesting the enzyme was relatively thermostable. It was strictly metal-dependent and required Mn^2+^ as optimum cofactor for activity. In addition, Mn^2+^ improved the structural stability during both heat- and urea-induced unfolding. Using circular dichroism measurements, the apparent melting temperature (*T*_m_) and the urea midtransition concentration (*C*_m_) of metal-free enzyme were 64.4°C and 2.68 M. By comparison, the Mn^2+^-bound enzyme showed higher *T*_m_ and *C*_m_ with 67.3°C and 5.09 M. The Michaelis-Menten constant (*K*_m_), turnover number (*k*_cat_), and catalytic efficiency (*k*_cat_/*K*_m_) values for substrate D-psicose were estimated to be 28.3 mM, 1826.8 s^−1^, and 64.5 mM^−1^ s^−1^, respectively. The enzyme could effectively produce D-psicose from D-fructose with the turnover ratio of 28%.

## Introduction

D-Psicose (D-ribo-2-hexulose or D-allulose), the C3 epimer compound of D-fructose, is considered as a kind of rare sugar, which was defined by the International Society of Rare Sugars (ISIR) as monosaccharides and their derivatives existing in nature in extremely small quantities (The 1^st^ International Symposium of ISRS, Takamatsu, Japan, 2002). It has 70% relative sweetness but only 0.3% energy of sucrose [1], and is suggested as an ideal sucrose substitute for food products [2]. It has important physiological functions, such as blood glucose suppressive effect [3], [4], reactive oxygen species scavenging activity [5], [6], and neuroprotective effect [7]. In addition, it can improve the gelling behavior and produces good flavor during food process [8], [9]. Importantly, it has been approved as “generally regarded as safe” (GRAS) by Food and Drug Administration (FDA) in Jun. 2012 (GRN No. 400), and has been allowed to be used as ingredient in a range of foods and dietary supplements. Commercial bioproduction of D-psicose may be realized through epimerization from D-fructose using D-tagatose 3-epimerase (DTEase) family enzymes, which have recently attracted attention of many researchers by its importance in application of rare sugar bioproduction [2].

Although the genes of DTEase family enzymes are widely predicted in various microorganisms, only a few of the enzymes have been conclusively characterized and identified through experimental evidence. Twenty years ago, DTEase was firstly characterized by Izumori et al. from *Pseudomonas cichorii*, showing C-3 epimerization activity of ketohexoses with the optimum substrate of D-tagatose [10]. Also, the *P. cichorii* DTEase was successfully used in D-psicose mass bioproduction [11], [12]. In 2006, the second enzyme with C-3 epimerization activity of ketohexoses was identified from *Agrobacterium tumefaciens*, and it was named D-psicose 3-epimerase (DPEase), due to its high substrate specificity for D-psicose [13]. Recently, another three DTEase family enzymes were characterized from *Rhodobacter sphaeroide*s SK011 (DTEase) [14], *Clostridium cellulolyticum* H10 (DPEase) [15], and *Ruminococcus* sp. 5_1_39BFAA (DPEase) [16], respectively.

The crystal structures of *A. tumefaciens* DPEase [17], *P. cichorii* DTEase [18], and *C. cellulolyticum* DPEase [19] have been identified. All the enzymes assemble into a tetramer and the subunits have around 295 amino acid residues with the molecular weight of approximately 32 kDa. They show the similar (β/α)_8_ TIM barrel structure and display a divalent metal coordinating site in the enzyme’s active center. Through multiple sequence alignment of reported DTEase family enzymes, they show relatively low similarity (20–60% identity) of amino acid sequence, but the amino acid residues responsible for metal coordination are strictly conserved in all the enzymes, suggesting that the divalent metal ion is possibly important to the structure and function of the enzyme as the cofactor. Interestingly, the abovementioned DTEase family enzymes show different requirement for metal cofactor to the activity and stability of the enzyme. *P. cichorii* DTEase does not require any cofactor for its activity, and even loses activity under some metal ions [20]. *R. sphaeroides* DTEase [14], *A. tumefaciens* DPEase [13], and *Ruminococcus* sp. DPEase [16] can display activity without metal ion, but their activity is remarkably enhanced by metal ions especially Mn^2+^. However, *C. cellulolyticum* DPEase is strictly metal-dependent and does not show activity in absence of metal ions [15]. *C. cellulolyticum* DPEase requires metal ion as essential cofactor to display activity and shows the maximal activity in the presence of Co^2+^. In addition, the Co^2+^ can significantly improve the thermostability of *C. cellulolyticum* DPEase [15].

In this study, the gene encoding the hypothetical protein CLOSCI_02528 with protein ID ZP_02432283 from the *Clostridium scindens* ATCC 35704 was cloned and overexpressed in *E. coli*. The hypothetical protein CLOSCI_02528 was identified as a new member of DTEase family enzymes. The enzyme was strictly metal-dependent like *C. cellulolyticum* DPEase, and its optimum substrate was D-psicose. The enzyme properties and kinetic parameters were determined, and compared to those of other DTEase family enzymes. Especially, the effect of metal ion on the activity and stability of the enzyme was studied in detail.

## Materials and Methods

### Chemicals and Reagents

The resin for protein purification, the Chelating Sepharose Fast Flow, was obtained from GE (Uppsala, Sweden). The pET-22b(+) expression vector was obtained from Novagen (Darmstadt, Germany). Electrophoresis reagents were purchased from Bio-Rad. Isopropyl β-D-1-thiogalactopyranoside (IPTG) and all chemicals used for enzyme assays and characterization were at least of analytical grade obtained from Sigma (St Louis, MO, USA) and Sinopharm Chemical Reagent (Shanghai, China).

### Gene Cloning and Protein Expression

The full-length nucleotide sequence of the gene encoding the hypothetical protein CLOSCI_02528 from the *C. scindens* ATCC 35704 was synthesized and incorporated with *Nde*I and *Xho*I sites in the 5′- and 3′-terminal of the gene, respectively, and then cloned into pUC57 vector by Shinegene Molecular Biothnology Co., Ltd (Shanghai, China). Then the plasmid was sub-cloned into pET-22b(+) vector with *Nde*I and *Xho*I sites, therefore, an in-frame fusion 6×histidine-tag sequence at the C-terminus was provided in the reconstructed plasmid, named by pET-cs-dpe. And the pET-cs-dpe was transformed into *E. coli* BL21(DE3).

The recombinant *E. coli* for protein expression was cultivated with shaking (200 rpm) in 400 ml of LB medium containing 50 µg/ml ampicillin at 37°C. When the OD_600_ reached 0.6, IPTG was added at 0.5 mM, and DPEase was induced and overexpressed at 30°C for 6 h, and harvested by centrifugation at 4°C for 10 min at 10,000* g*. The enzyme was expressed as 6×histidine-tagged fusion protein, which was available for affinity chromatography.

### Protein Purification

Four gram of wet cell pellets were resuspended in 20 mL of lysis buffer (50 mM Tris-HCl, 100 mM NaCl, pH 7.5) and disrupted by sonication at 4°C for 6 min (pulsations of 3 s, amplify 90) using a Vibra-Cell™ 72405 Sonicator. The lysate was centrifuged at 20,000 *g* for 30 min at 4°C. Then, the 6×histidine-tagged fusion enzyme was purified by nickel-affinity chromatography (GE Healthcare) according to manufacturer’s protocol (Instructions 71-5001-87 AE; GE Healthcare). The 15 mL of supernatant was loaded onto Chelating Sepharose Fast Flow resin column (1.0 cm×10.0 cm), previously chelating NiSO_4_ and equilibrated with a binding buffer (50 mM Tris-HCl, 500 mM NaCl, pH 7.5). After washing with the washing buffer (50 mM Tris-HCl, 500 mM NaCl, 50 mM imidazole, pH 7.5) to remove unbound fractions, the DPEase was eluted from the column with an elution buffer (50 mM 50 mM Tris-HCl, 500 mM NaCl, 500 mM imidazole, pH 7.5). The active fractions were pooled and dialyzed overnight against 50 mM Tris-HCl buffer (pH 7.5) containing 10 mM ethylenediamine tetraacetic acid (EDTA) at 4°C. Subsequently, the enzyme was dialyzed against 50 mM EDTA-free Tris-HCl buffer (pH 7.5) using a 10-kDa cut-off membrane (Millipore). Protein concentration was determined by the method of Bradford using bovine serum albumin as a standard. SDS-PAGE was carried out according to Laemmli. Gels (12% w/v polyacrylamide) were stained with Coomassie Brilliant Blue and destained with an aqueous mixture of 10% (v/v) methanol/10% (v/v) acetic acid.

### Enzyme Assay

The activity was measured by the determination of the amount of produced D-psicose from D-fructose. The reaction mixture of 1 mL contained D-fructose (50 g/L), Tris-HCl buffer (50 mM, pH 7.5), 1 mM Mn^2+^, and 1 µM enzyme. The reaction was carried out at 60°C for 10 min, and was terminated by boiling for 5 min. The generated D-psicose was determined by the HPLC method. One unit of enzyme activity was defined as the amount of enzyme catalyzing the formation of 1 µmol D-psicose per min.

### Effect of Temperature, pH, and Metal Ions

The optimum temperature of enzyme activity was measured by assaying the enzyme samples over the range of 35–75°C for 2 min. Two buffer systems, sodium phosphate (50 mM, pH 5.5–7.0) and Tris-HCl (50 mM, pH 7.5–9.0), were used for measuring the optimum pH of enzyme activity. The thermal stability of the enzyme was studied by incubating the enzyme in Tris-HCl buffer (50 mM, pH 7.5) at various temperatures. At given time intervals, samples were withdrawn and the residual activity was measured under standard assay conditions. To determine the pH stability, the enzyme was incubated at pH 5.5–9.0 at 4°C for up to 2 h, and the remaining enzyme activity was measured at time intervals under standard assay conditions. To determine the effects of cations, the samples were incubated in the presence of the different divalent metal ions at 1 mM: MnCl_2_, CoCl_2_, FeCl_2_, NiCl_2_, and MgCl_2_, respectively.

### Determination of Kinetic Parameters

Kinetic parameters of *C. scindens* DPEase were determined in 50 mM Tris-HCl buffer (pH 7.5) containing 1 mM Mn^2+^ and D-pricose, D-fructose, or D-tagatose as the substrate from 5 to 200 mM. Kinetic parameters, such as Michaelis-Menten constant (*K*_m_) and turnover number (*k*_cat_) values for substrates were obtained using the Lineweaver-Burk equation and quantification of enzyme concentration.

### Circular Dichroism Measurements and Protein Unfolding Analysis

Circular dichroism (CD) measurements were carried out with a Jasco J-810 spectropolarimeter with a Peltier temperature-controlled cuvette holder. For thermal unfolding, protein concentration was set as 0.4 mg/ml in 50 mM Tris–HCl, and protein unfolding was monitored by CD ellipticity at 220 nm from 40 to 86°C at a heating rate of 1°C/min. Samples were overlaid with mineral oil and the lids of cuvette were sealed with paraffin tape to prevent evaporation. For urea-induced unfolding, protein samples (0.5 mg/ml in 50 mM Tris–HCl) were diluted in urea (0.32 mM increments up to a final urea concentration between 0–6 M), pre-equilibrated for 2 h at room temperature, and the change in the folded fraction was monitored by a CD signal at 220 nm at 25°C.

The ratio of denatured and total protein in the transition range, *f*_d_ = (ε_N_–ε)/(ε_N_–ε_U_), was calculated from their CD signal relative to the signal of the native and unfolded base line ε_N_ and ε_U_. Melting concentration (*T*_m_) and urea mid-transition concentration (*C*_m_) were defined as the transition mid-point of thermal and urea-induced protein unfolding, respectively.

### HPLC Measurements of Monosaccharides

The concentrations of D-fructose and D-psicose were analyzed by HPLC equipped with a refractive index detector and a Ca^2+^-carbohydrate column (Waters Sugar-Pak 1, Waters Corp., Milford, MA, USA). The monosaccharides were separated by isocratic elution in water, with column temperature at 85°C and flow rate at 0.4 mL/min.

## Results and Discussion

### Amino Acid Sequence Alignment of DTEase Family Enzymes

**Figure 1:**
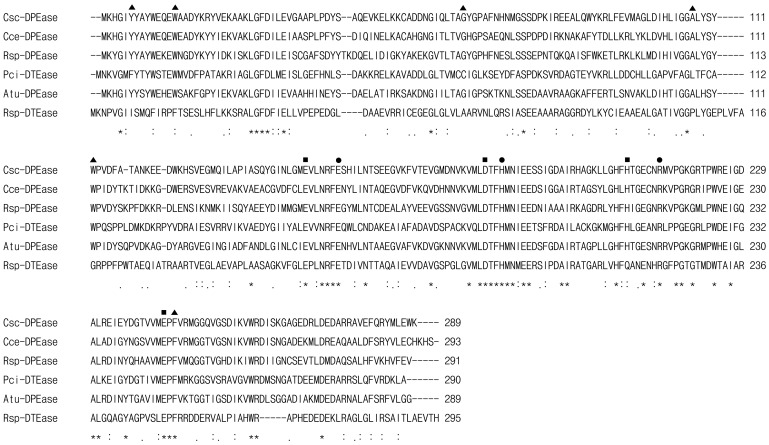
Multiple sequence alignment of DTEase family enzymes and their homologs. Amino acid sequence for DPEase from *C. scindens* 35704 (Csc-DPEase; GeneBank accession No: CLOSCI_02528) was aligned with *C. cellulolyticum* H10 (Cce-DPEase; ACL75304), *Ruminococcus* sp. 5_1_39BFAA (Rsp-DPEase; ZP_04858451), *P. cichorii* DTEase (Pci-DTEase; BAA24429), *A. tumefaciens* DPEase (Atu-DPEase; AAL45544), and *R. sphaeroides* DTEase (Rsp-DTEase; ACO59490). The alignment was performed using ClustalW2 program (http://www.ebi.ac.uk/Tools/clustalw2/index.html). Amino acid residues that are identical in all the displayed sequences are marked by asterisks (*), strongly conserved or weakly conserved residues are indicated by colons (:) or dots (.), respectively. The symbol ▪, •, and ▴ represented the residues involved in the metal coordinating site, those responsible for the interaction between the enzyme and O1, O2, and O3 of D-fructose, and those providing a hydrophobic environment around the substrate around the O4, O5, and O6 of D-fructose, respectively (according to the crystal structures of *C. cellulolyticum* DPEase, *A. tumefaciens* DPEase, and *P. cichorii* DTEase).

Compared to the reported microbial DTEase family enzymes, the DPEase from *C. scindens* 35704 (hypothetical protein CLOSCI_02528 with protein ID ZP_02432283) was found relatively homologous in amino acid sequence with the family enzymes from *C. cellulolyticum* H10, *A. tumefaciens*, *Ruminococcus* sp. 5_1_39BFAA, *P. cichorii*, and *R. sphaeroide*s SK011, with 62%, 58%, 52%, 38%, and 29% identity, respectively. Although the homology among the family enzymes was not high (20–60%) in amino acid sequence, they had highly similarities in the key amino acid residues of active sites, metal coordinating sites and substrate combinant binding sites (Fig. 1). From the known structure information of *A. tumefaciens* DPEase [17], *P. cichorii* DTEase [18], and *C. cellulolyticum* DPEase [19], the metal coordinating sites were predicted consisting of Glu149, Asp182, His208, and Glu243 in *C. scindens* DPEase, and were strictly conserved in all DTEase family enzymes. The amino acid residues Glu156, His186, and Arg215, which were responsible for the interaction between the enzyme and O1, O2, and O3 of substrate, were also conserved in the family enzymes. However, interestingly, as for the residues providing a hydrophobic environment around the substrate, which consisted of Tyr6, Try14, Gly65, Ala107, and Phe245, *C. scindens* DPEase showed absolute identity with all reported DPEases, but was significantly different with the DTEases (Fig. 1). It was probably caused by the substrate binding sites composed by different amino acid residues in the structure.

### Cloning, Expression, and Purification of *C. scindens* DPEase

The *C. scindens* 35704 genomic sequence was completed and deposited as GenBank accession number NZ_DS499706. According to the genome sequencing, the nucleotide sequence of the gene coding hypothetical protein CLOSCI_02528 (characterized as DPEase) was given as Gene ID 167760156. This DPEase-coding gene was obtained by the full-length gene synthesis, instead of the PCR amplification *in vitro* using genome as DNA template. The coding gene sequence was intentionally incorporated with *Nde*I and *Xho*I sites in the 5′- and 3′-terminal of the gene, respectively, and then was cloned into pET-22b(+) vector with *Nde*I and *Xho*I sites, therefore, an in-frame fusion 6×histidine-tag sequence at the C-terminus was provided in the reconstructed plasmid, named by pET-cs-dpe. This allowed for a generic single step purification of the recombinant DPEase using nickel-affinity column chromatography.

**Figure 2:**
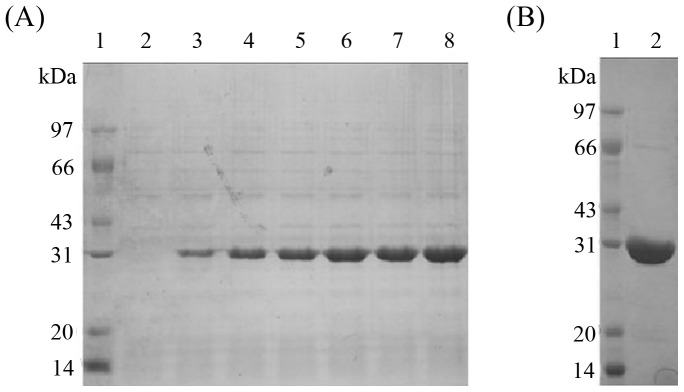
SDS-PAGE analysis of proteins stained with Coomassie blue. A represented the SDS-PAGE of whole-cell protein. Lane 1, protein marker; lane 2, the cell without IPTG induction; and lane 3–8, the cell induced by IPTG for 1–6 h. B showed the SDS-PAGE analysis of the purified recombinant *C. scindens* DPEase (lane 2).

The pET-cs-dpe was then introduced into the expression host, *E. coli* BL21(DE3), to overexpress DPEase by IPTG induction. SDS-PAGE analyses on the extracts of *E. coli* BL21(DE3) harboring pET-Csc-dpe induced by IPTG revealed the presence of large amounts of protein around 31 kDa, compared to that of the control *E. coli* BL21(DE3) cells harboring plasmid pET-22b(+); and the molecular weight was in agreement with the predicted molecular mass for the DPEase protein, suggesting the DPEase was overexpressed by IPTG induction (Fig. 2A). After IPTG induction, the *E. coli* cells were harvested by centrifugation and disrupted by sonification. Cell debris was removed by centrifugation at 4°C and 20,000 g for 20 min. The supernatant was subjected to the Ni^2+^-Chelating Sepharose Fast Flow affinity column, and the recombinant DPEase fused by C-terminal 6×histidine-tag was purified to electrophoretic homogeneity with a single band on SDS-PAGE (Fig. 2B). The molecular weight of the purified *C. scindens* DPEase was estimated to be approximately 32 kDa by SDS-PAGE, however, the gel filtration experiment data showed the molecular weight of native enzyme was approximately 125 kDa, suggesting the enzyme was a tetramer with four identical subunits. The tetramer manner of subunits assembly was also characterized in other DTEase family enzymes, such as *A. tumefaciens* DPEase [13], *C. cellulolyticum* DPEase [15], and *Ruminococcus* sp. DPEase [19]; however, the *R. sphaeroide*s SK011 DTEase [14] and *P. cichorii* DTEase were determined as dimers [21].

### Effects of Temperature and pH on *C. scindens* DPEase

**Figure 3:**
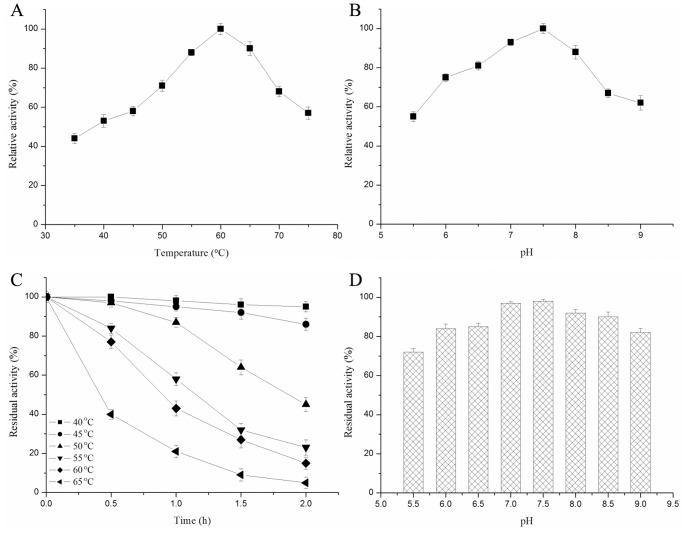
Enzymatic properties of *C. scindens* DPEase. Panel A and B showed the effect of pH and temperature on the activity of *C. scindens* DPEase. C and D represented the effect of temperature and pH on the stability of *C. scindens* DPEase. The thermal stability was investigated by exposing the enzyme at different temperature for different time intervals at pH 7.5. The pH stability was analyzed by pre-incubating the enzyme buffers of different pH values at 4°C for 2 h and measuring the remaining activity at 60°C and pH 7.5. Values are means of three replications ± standard deviation.

The temperature and pH profiles for *C. scindens* DPEase activity were shown in Fig. 3A and 3B, respectively. The enzyme activity was detected in presence of 1 mM Mn^2+^, because it displayed null activity without metal ions. The optimum temperature and pH for enzyme activity are 7.5 and 60°C. Comparison of biochemical properties of *C. scindens* DPEase and other reported DTEase family enzymes was shown in Table 1. The optimum temperature of the known DTEase family enzymes is the range of 40–60°C and the optimum pH is all weakly alkaline ranged from 7.5 to 9.0.

**Table 1:** Comparison of biochemical properties and kinetic parameters of DTEase family enzymes.

Strains	*C. scindens* a	*C. cellulolyticum* [15]	*Ruminococcus* sp.[16]	*A. tumefaciens* [13]	*P. cichorii* [20]	*R. sphaeroides* [14]
Optimum temperature (°C)	60	55	60	50	60	40
Optimal pH	7.5	8.0	7.5–8.0	8.0	7.5	9.0
Metal ion requied	Mn^2+^	Co^2+^	None	Mn^2+^	None	Mn^2+^
Half-life at 50°C (min)	108	>120	>240	63.5	NR	Approximately 60
Substrate with highestspecificity	D-Psicose	D-Psicose	D-Psicose	D-Psicose	D-Tagatose	D-Fructose
*k*_cat_ (min ^−1^)	1,827±136 (D-Psicose)350±26 (D-Fructose)44.2±3.8 (D-Tagatose)	3,243.5 (D-Psicose)3,354.5 (D-Fructose) 184.8 (D-Tagatose)	2,427 (D-Psicose)3,562 (D-Fructose)109 (D-Tagatose)	2381 (D-Psicose)2068 (D-Fructose)270 (D-Tagatose)	NR^b^	NR
*K*_m_ (mM)	28.3±3.2 (D-Psicose)40.1±2.5 (D-Fructose)175.7±19.3 (D-Tagatose)	17.4 (D-Psicose)53.5 (D-Fructose)244 (D-Tagatose)	48 (D-Psicose)216 (D-Fructose)231 (D-Tagatose)	12 (D-Psicose)24 (D-Fructose)762 (D-Tagatose)	55 (D-Tagatose)	NR
*k*_cat_/*K*_m_(mM^−1^ min ^−1^)	64.5±4.8 (D-Psicose)8.72±0.63 (D-Fructose)0.25±0.02 (D-Tagatose)	186.4 (D-Psicose)62.7 (D-Fructose)0.75 (D-Tagatose)	51 (D-Psicose)16 (D-Fructose)0.47 (D-Tagatose)	205 (D-Psicose)85 (D-Fructose)0.35 (D-Tagatose)	NR	NR
Equilibrium ratio between D-psicose and D-fructose	28∶72 (50°C)	32∶68 (55°C)	28∶72 (60°C)	32∶68 (30°C)33∶67 (40°C)	20∶80 (30°C)	23∶77 (40°C)

a The kinetic parameters of *C. scindens* DPEase are means of three replications ± standard deviation.

b NR, not reported.

*C. scindens* DPEase retained 95 and 86% of its initial activity after 2 h of exposure at 40 and 45°C (Fig. 3C), which indicated that the enzyme was relatively stable below 50°C. The half-life of the enzyme at 50°C was approximately 108 min, which was longer than that of, *A. tumefaciens* DPEase (63.5 min) [13] and *P. cichorii* DTEase (approximately 60 min) [20], but shorter than that of *C. cellulolyticum* DPEase [15] and *Ruminococcus* sp. DPEase [16]. Like other wild DTEase family enzymes, *C. scindens* DPEase was easily deactivated under exposure at higher temperatures of more than 55°C. When incubated at 60°C, the half-life of *C. scindens* DPEase was around 50 min, which was shorter than that of *Ruminococcus* sp. DPEase (1.6 h) [16], but longer than other wild DTEase family enzymes. In addition, more than 80% activity of the purified *C. scindens* DPEase was retained pH 6.0–9.0 after incubation at 4°C for 2 h (Fig. 3D), indicating that the *C. scindens* DPEase was relatively stable under neutral and weakly alkaline conditions.

Thermostability is an important property of DTEase family enzymes to realize the industrial enzymatic production of D-psicose. Some DTEase family enzymes could be more thermostable in presence of metal ion cofactor, such as *C. cellulolyticum* DPEase [15] and *Ruminococcus* sp. DPEase [16]. The thermostability also could be improved through the molecular modification. Choi et al. constructed the I33L S213C double-site variant of *A. tumefaciens* DPEase, and the variant enzyme showed significant increases in optimal temperature, half-life, and melting temperature, compared with the wild-type enzyme; and the variant enzyme’s half-life at 50°C reached 1,853 min, which was 29.9-fold of that of the wild-type enzyme [22].

### Effects of Metal Ions on *C. scindens* DPEase Activity

*C. scindens* DPEase displayed null activity in absence of metal ion, and only had catalysis activity when adding divalent metal ion. Also, even in presence of metal ion, the enzyme would lose all activity when adding EDTA as metal ion chelating agent. Therefore, the *C. scindens* DPEase was a kind of strictly metal-dependent enzyme, which is similar to *C. cellulolyticum* DPEase, but different with other DTEase family enzymes [15].

To investigate the effect of metal ions, the relative activity of *C. scindens* DPEase was detected in presence of different divalent metal ions, which were added at the final concentration of 1 mM. *C. scindens* DPEase showed a maximal activity in the presence of Mn^2+^; however, when Mn^2+^ was replaced with Co^2+^, Fe^2+^, Ni^2+^, and Mg^2+^, the enzyme activity was reduced to 92%, 70%, 69%, and 41% of that in presence of Mn^2+^, respectively. By comparison, *C. cellulolyticum* DPEase is also metal-dependent but with optimum metal ion of Co^2+^ [15]; *P. cichorii* DTEase does not require any cofactor for its activity [20]; *Ruminococcus* sp. DPEase [16], *A. tumefaciens* DPEase [13], and *R. sphaeroides* DTEase [14] can display activity without metal ion, but their activity is significantly enhanced by metal ions especially Mn^2+^ or Co^2+^.

**Figure 4:**
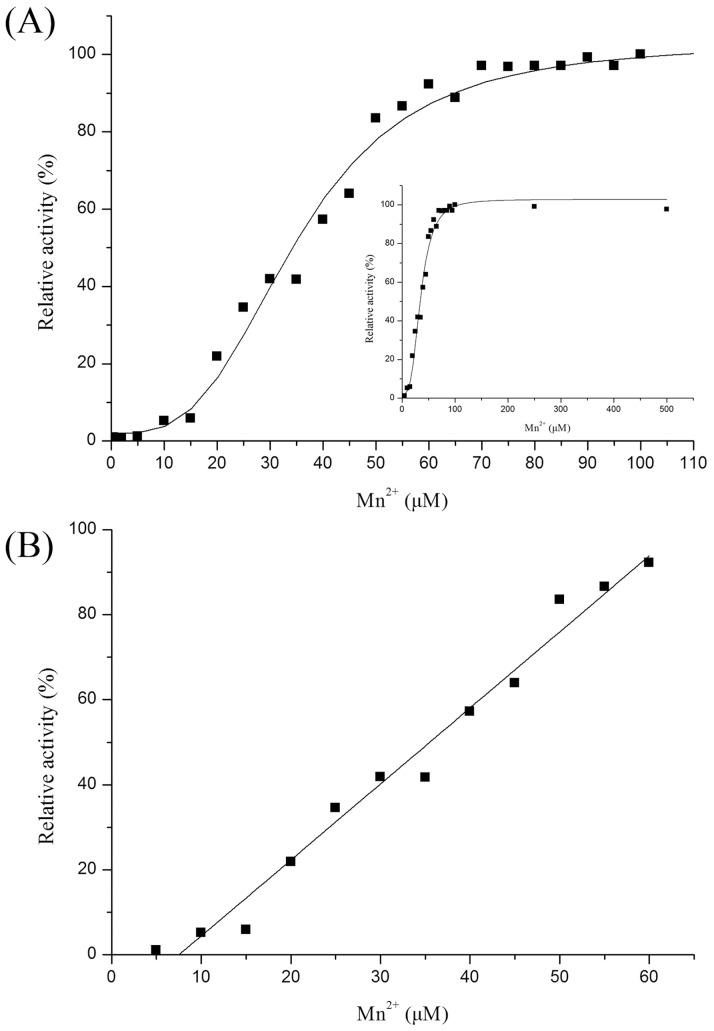
Effect of Mn^2+^ on the catalysis activity of *C. scindens* DPEase. Panel A showed the relative activities in conditions of 0–100 µM Mn^2+^. The curve in the inset represented the effect of Mn^2+^ with broad concentration range from 0 to 600 µM. And panel B represented the linear range of Mn^2+^ concentration effect on the enzyme activity. The experiments were developed with 1 µM *C. scindens* DPEase.

In addition, the effect of metal cofactor concentration on catalytic activity of metal-dependent DPEase was also investigated. The Mn^2+^ concentration titration on catalytic activity with 1 µM *C. scindens* DPEase was developed, and the overall results of the Mn^2+^ concentration effect on activity was like S-curve, and different with general first order reaction model (Fig. 4A). The activity could not be detected when the Mn^2+^ concentration was below 5 µM, probably because the enzyme needed a certain amount of Mn^2+^ concentration to effectively bind the metal cofactor. Then, the relative activity increased linearly with the addition of metal ion with between 5 and 60 µM, which accorded with first order reaction (Fig. 4B). When the Mn^2+^ concentration was more than 60 µM, the relative activity tended to be stationary, probably because the binding of enzyme to metal ion was close to saturation (Fig. 4A). The similar result was reported on another metal-dependent enzyme, *Geobacillus stearothermophilus* L-arabinose isomerase, which also displayed null activity without metal ions, but rapidly restored catalytic activity by adding trace amount of Mn^2+^ [23]. Interestingly, for aldose isomerase or ketose epimerase having metal coordinating region in structure, some enzyme are metal-dependent and must require metal cofactor to display activity [15], [23], [24]; some not only do not require metal ions but also could not be activated by metal ions [20], [25], [26]; and other could display catalytic activity without metal ion, however, their activity could be remarkably enhanced by metal ions [13], [14], [16]. The mechanism of different metal independence of these enzymes having metal coordinating region may be fascinating topic worthy of deep investigation.

### Effects of Mn^2+^ on Structural Stability of *C. scindens* DPEase

**Figure 5:**
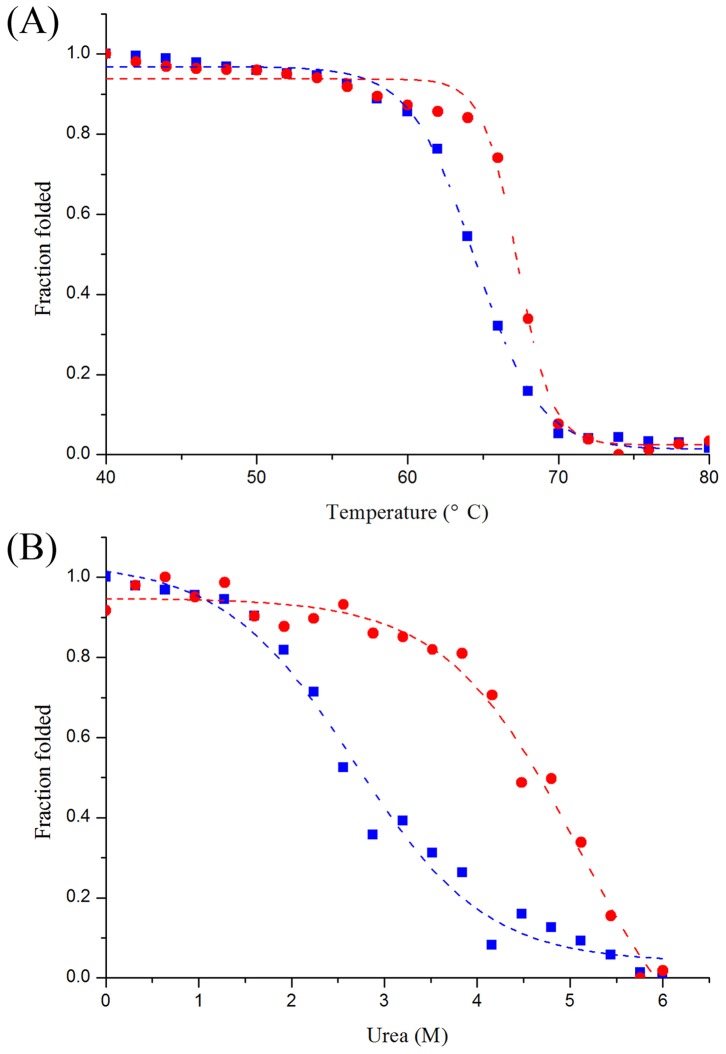
Thermal (A) and urea-induced (B) unfolding of *C.* *scindens* DPEase. The symbols ▪ and • represented the unfolding curves of *C. scindens* DPEase in the absence and presence of Mn^2+^, which were monitored by CD. The CD measurements and unfolding analysis procedures were shown in Materials and Methods.

To study the effect of Mn^2+^ on thermostability based on the structural aspects, thermal unfoldings of both enzymes in the absence and the presence of Mn^2+^ were performed using CD. Shown in Fig. 5A, metal-free *C. scindens* DPEase had the apparent melting temperature (*T*_m_) of 64.4°C, whereas the metal-bound enzyme showed higher *T*_m_ of 67.3°C. Therefore, it was suggested that the metal ion was important for *C. scindens* DPEase with respect to the unfolding and structural stability during thermal denaturation. Similar result about the improvement effect of metal ion on the thermostability was reported on the metal-dependent *G. stearothermophilus* L-arabinose isomerase, in which the *T*_m_ of metal-bound enzyme was 6°C higher than that of the metal-free enzyme [23].

In addition to the thermal unfolding, the urea-induced unfoldings of both metal-free and metal-bound *C. scindens* DPEases were also studied using CD. According to the urea-induced denaturation curves (Fig. 5B), the urea midtransition concentration (*C*_m_) was derived, and the *C*_m_ of metal-bound *C. scindens* DPEase (5.09 M) was much higher than that of metal-free enzyme (2.68 M), suggesting that the metal ion effectively improved the structural stability of enzyme against the protein denaturant urea. Therefore, the metal ion not only was the essential cofactor for *C. scindens* DPEase to have the catalysis activity, but also could improve the structural stability during both heat- and urea-induced unfolding.

### Substrate Specificity, Enzyme Kinetics, and Equilibrium Ratio

The optimum substrate of *C. scindens* DPEase was D-psicose, and the substrate specificity decreased in the following order: D-fructose, D-tagatose, and D-sorbose. As other known DTEase family enzymes, *C. scindens* DPEase could not catalyze D-fructose-6-phosphate or D-ribulose-5-phosphate. *C. scindens* DPEase had the similar substrate specificity with *Ruminococcus* sp. DPEase [16], *C. cellulolyticum* DPEase [14], and *A. tumefaciens* DPEase [13], showing the optimum substrate of D-psicose; however, the optimum substrate of *P. cichorii* DTEase [20] and *R. sphaeroide*s DTEase [14] was D-tagatose and D-fructose, respectively.

The Michaelis-Menten constant (*K*_m_), turnover number (*k*_cat_), and catalytic efficiency (*k*_cat_/*K*_m_) values of *C. scindens* DPEase for substrate D-psicose were estimated to be 28.3 mM, 1826.8 min^−1^, and 64.5 mM^−1^ min^−1^, respectively. And the *k*_cat_/*K*_m_ for D-psicose was much higher than that for other substrates, and was 7.4-fold and 258-fold of that for D-fructose and D-tagatose, respectively. These data are very similar to those of *Ruminococcus* sp. DPEase [16], *C. cellulolyticum* [15] and *A. tumefaciens* DPEases [13], showing these four DPEases had the same substrate specificity (Table 1).

The equilibrium ratio between D-psicose and D-fructose of *C. scindens* DPEase was measured to be 28∶72, which was higher than that of *P. cichorii* DTEase (20∶80) [20] and *R. sphaeroides* DTEase (23∶77) [14], but lower than that of *C. cellulolyticum* DPEase (32∶68) [15] and *A. tumefaciens* DPEase (33∶67) [13] (Table 1). In addition, *C. scindens* DPEase could epimerize D-tagatose into D-sorbose, with the turnover ratio of 8.6%.

In conclusion, the noncharacterized gene CLOSCI_02528 from *C. scindens* ATCC 35704, encoding the hypothetical protein ZP_02432283, was cloned and expressed in *E. coli*, and the protein was characterized as a member of DTEase family enzymes with the optimum substrate of D-psicose. The enzyme showed maximum activity at pH 7.5 and 60°C. It was strictly metal-dependent and required Mn^2+^ as optimum cofactor for activity. In addition, Mn^2+^ improved the structural stability during both thermal and urea-induced unfolding. The *k*_cat_/*K*_m_ for D-psicose and D-fructose was estimated to be 64.5 and 8.72 mM^−1^ min^−1^, respectively. The equilibrium ratio between D-psicose and D-fructose of the enzyme was 28∶72.
