# Correction: Characterization of a Novel Metal-Dependent D-Psicose 3-Epimerase from *Clostridium scindens* 35704

**DOI:** 10.1371/annotation/4bc8d881-0bee-4ccd-8c1a-f73833589134

**Published:** 2014-01-06

**Authors:** Wenli Zhang, Dan Fang, Qingchao Xing, Leon Zhou, Bo Jiang, Wanmeng Mu

Figure 2 in the article, which reports the overexpression and purification of Clostridium scindens DPEase (previously named D-tagatose 3-epimerase) is duplicate from the authors' publication in Food and Fermentation Industries below:

Xing Q, Mu W, Jiang B, Zhou L, Zhang T (2011) Cloning, expression, purification and characterization of D-tagatose 3-epimerase from Clostridium scindens ATCC 35704. Food Ferment Ind 37(8): 6-10 (in Chinese).

This publication and the duplication of the figure should have been acknowledged in the article and the authors apologize for this oversight.

Figure 2 in the PLOS ONE article is reproduced under a Creative Commons Attribution License with permission by Food and Fermentation Industries, the authors thank the journal Food and Fermentation Industries for providing this permission.

The publication by Xing et al. above reported the gene cloning, overexpression and purification of the putative D-tagatose 3-epimerase from C. scindens 35704. The enzyme showed effective epimerization activity between D-tagatose and D-sorbose and also between D-fructose and D-psicose, but the relative activities and kinetic parameters toward various substrates were not measured and the enzymatic properties were not evaluated.

The PLOS article is focused on an in-depth analysis of the enzyme's properties including the effects of metal, temperature and pH, pH stability, and thermostability; we measured the relative activities and kinetic parameters toward various substrates of the enzyme and studied the effects of metal on the structural stability against both temperature and denaturant urea. The putative D-tagatose 3-epimerase from C. scindens 35704 was then renamed D-psicose 3-epimerase, because the enzyme showed substrate specificity and the highest catalytic efficiency toward D-psicose (Table 1).

The authors would also like to clarify that Figure 1 in this article is similar but different to Figure 1 in the previous publication by Mu et al. (J Agric Food Chem, 2011, 59:7785-7792, reference 15). These two figures described the multiple sequence alignment of reported DTEase family enzymes. Each figure presented the latest comparison data of all the already-characterized DTEase family enzymes. Figure 1 in the previous article by Mu et al. presented a comparison of the sequences for C. cellulolyticum DPEase, A. tumefaciens DPEase, P. cichorii DTEase, R. sphaeroides DTEase, and T. maritima TM0416p. Figure 1 in PLOS ONE article includes the latest C. scindens DPEase and Ruminococcus sp. DPEase , while T. maritima TM0416p (not DTEase family enzyme) was removed. 

